# Cases of Common Carotid Artery Pseudoaneurysm Treated by Stent Graft

**DOI:** 10.1155/2012/674827

**Published:** 2012-05-08

**Authors:** Hee Ok Kim, Yong Bae Ji, Seung Hwan Lee, Cheolkyu Jung, Kyung Tae

**Affiliations:** ^1^Department of Otolaryngology-Head and Neck Surgery, College of Medicine, Hanyang University, Seongdong-gu, Seoul 133-792, Republic of Korea; ^2^Department of Radiology, Seoul National University Bundang Hospital, Seongnam-si, Gyeonggi-do 463-707, Republic of Korea

## Abstract

Common carotid artery (CCA) pseudoaneurysms are rare and potentially lethal, and adequate treatment is warranted in order to prevent rupture or neurologic sequelae. The causes of CCA pseudoaneurysm include blunt or penetrating trauma, infection, and vasculitis, as well as iatrogenic and unknown causes. Previously, surgery was the standard treatment for pseudoaneurysm. However, endovascular surgical approaches such as stent graft or coiling have become effective alternatives with minimal morbidity and high success rates. Here, we report two cases of CCA pseudoaneurysms that were successfully treated by stent graft and review the current literature.

## 1. Introduction

A pseudoaneurysm is defined by the loss of integrity of all three layers of the arterial wall. This finding distinguishes it from a true aneurysm, in which the arterial wall is intact but expanded [1]. In comparison to other locations, pseudoaneurysms of the carotid artery are quite rare and are almost always the result of blunt or penetrating trauma [2].

Common carotid artery (CCA) pseudoaneurysm is usually discovered as pulsate neck mass. The neurological deficit due to compression of nerve can also be seen. The intracranial occlusion or intracranial embolus can occur due to thrombus. This may lead to rupture; the results of which can be fatal, so immediate treatment is necessary [3].

The standard treatment for CCA pseudoaneurysm was surgical repair including ligation of the carotid artery with or without bypass procedure and arterial reconstruction. However, in recent years to avoid surgical morbidity, endovascular intervention has been the effective alternatives in the treatment of pseudoaneurysm [2]. We report here on two cases of CCA pseudoaneurysm that was successfully treated by stent graft.

## 2. Case  1

A 26-year-old male patient was admitted to our hospital with a pulsatile mass in the right side of his neck that had been present for one week. He had no history of neck trauma. The patient had been diagnosed with Behcet's disease two years prior to admission to the hospital, suffered from deep vein thrombosis related to his Behcet's disease, and was being treated with sulfasalazine, warfarin, and prednisolone.

**Figure 1:**
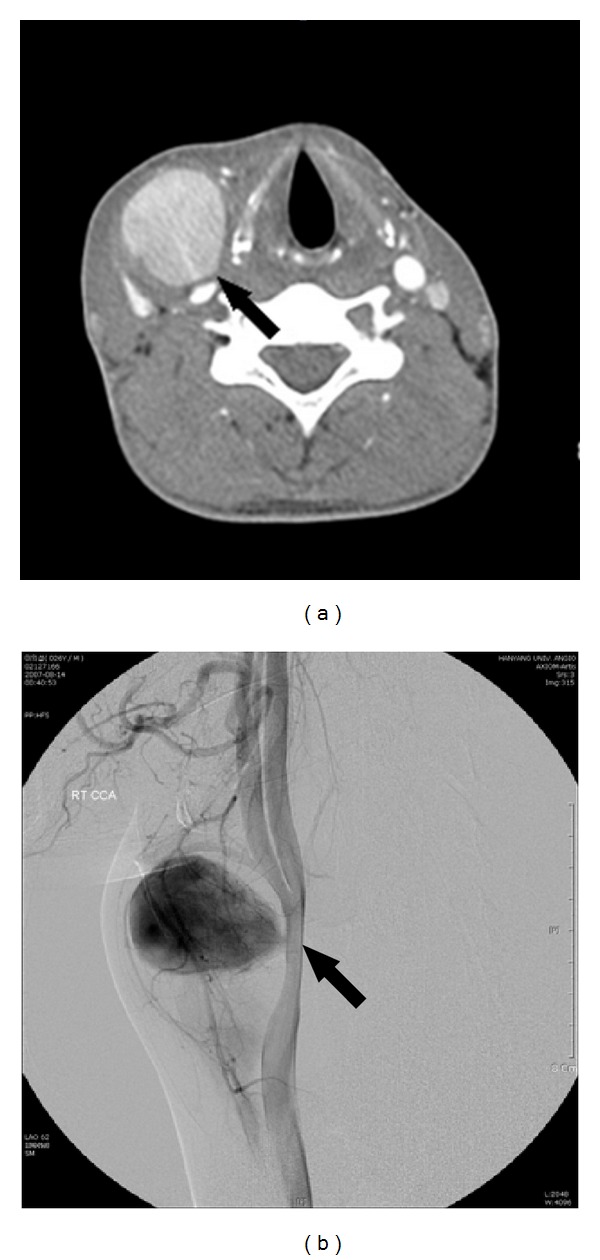
(a) Contrast-enhanced CT angiogram demonstrating a 3 × 3 cm sized pseudoaneurysm (arrow) at the right common carotid artery. (b) Right carotid angiogram demonstrating a pseudoaneurysm (arrow) arising from the right common carotid artery.

Physical examination revealed an approximately 3 × 3 cm sized painless, pulsatile mass on the right neck. The neurologic examination was normal, and his vital signs were stable. A computed tomography (CT) scan revealed a 3 × 3 cm sized well-enhanced CCA pseudoaneurysm (Figure 1(a)). An angiogram showed a pseudoaneurysm just at the level of the bifurcation of the right CCA (Figure 1(b)). An intracranial angiogram showed collateral blood flow from the left carotid artery through the anterior communication artery during compression of the right CCA.

**Figure 2:**
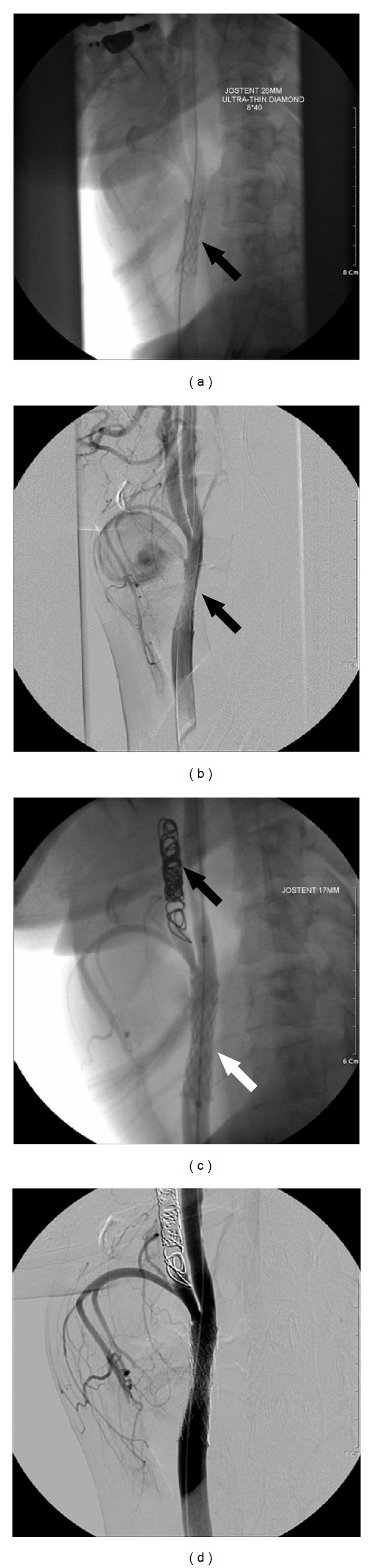
Intraoperative fluoroscopic image: (a) the Jostent was deployed at the right common carotid artery (black arrow). (b) Some extravasation of contrast material was noted at the pseudoaneurysm sac after stent deployment (black arrow). (c) The right external carotid artery was trapped by fibered coil (black arrow), and another Jostent (white arrow) was deployed in the right common carotid artery. (d) No contrast material was observed within the pseudoaneurysm after stent graft placement.

We decided to treat the patient with a stent graft, which first involved placement of a 0.0035 inch Terumo guide wire (Terumo, Tokyo, Japan) via the femoral approach into the right internal carotid artery, passing the right CCA. A JOSTENT (length 28 mm; diameter 4–9 mm; JOMED, Rangendingen, Germany) was then expanded in the CCA using an angioplasty balloon catheter (Ultra-Thin Diamond, 8 × 4 mm, Medi-Tech, Watertown, MA, USA). Due to insufficient support of the guide wire, the CCA defect was not completely covered by the stent, and a subsequent angiogram revealed contrast material entering into the pseudoaneurysm sac. Reinsertion of a stent was planned; however, in order to completely occlude the defective area, the stent was placed over both the CCA and the internal carotid artery (ICA). Because this procedure could lead to backflow through the external carotid artery (ECA), a coil was first used to block the ECA. Next, a JOSTENT (length 17 mm; diameter 4–9 mm; JOMED, Rangendingen, Germany) was placed into the ECA. After reinsertion of the stent, an angiogram showed that there was no longer contrast material within the pseudoaneurysm sac (Figure 2).

Several minutes after placing the stent graft, the patient complained of a sudden headache. An intracranial angiogram revealed thrombi in the middle cerebral artery. Immediately, an injection of nonpeptide Gp llb/llla antagonist was administered to the middle cerebral artery. After 15 minutes, an angiogram showed improved arterial flow and indicated that the thrombi had been resolved. The patient did not complain of subsequent headaches. Follow-up neurologic examination and a brain CT scan showed no further abnormal signs or findings. A total of seven months after placement of the stent graft the pseudoaneurysm was no longer visible on a CT scan, and blood flow of the stent area was patent.

## 3. Case  2

A 64-year-old female patient was admitted to our hospital with a suddenly enlarged pulsating mass of the right neck. The neck mass had developed two days prior to admission to the hospital. The patient had no history of neck trauma, surgery, or interventional procedure. She had been previously diagnosed with chronic obstructive pulmonary disease (COPD) and took regular medication to treat the disease. Physical examination revealed an approximately 3 × 4 cm sized painless, pulsating mass on the right side of the patient's neck with no neurological symptoms. The patient's vital signs were stable.

**Figure 3:**
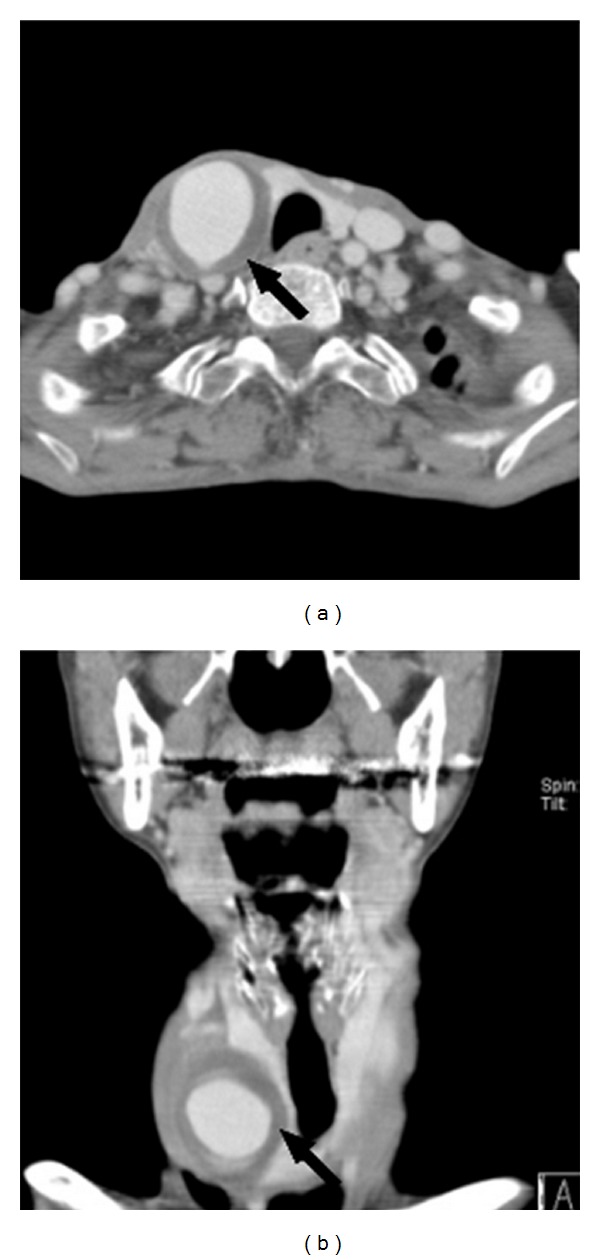
((a) and (b)) Contrast-enhanced CT angiograms demonstrating a 2.5 × 3.4 cm sized thrombosed pseudoaneurysm (arrow) in the right common carotid artery.

**Figure 4:**
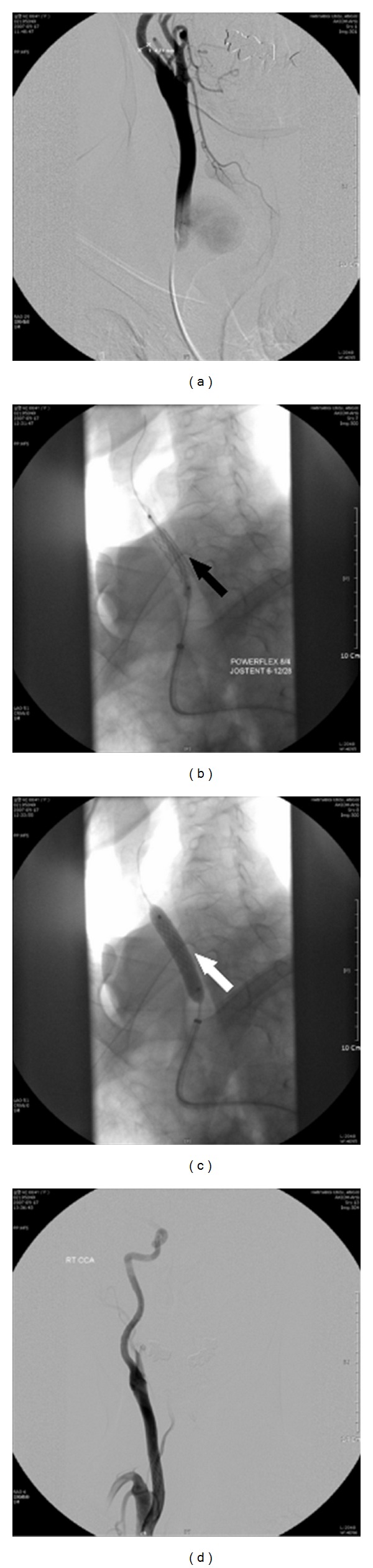
Intraoperative fluoroscopic image: (a) a right carotid angiogram demonstrating a pseudoaneurysm arising from the proximal common carotid artery. ((b) and (c)) The Jostent (black arrow) was deployed at the center of the fistula tract, and full dilatation was performed (white arrow). (d) The angiogram demonstrated a lack of contrast filling of the pseudoaneurysm after stent graft placement.

A CT scan revealed a 2.5 × 3.4 cm sized well-enhanced CCA pseudoaneurysm (Figure 3). The carotid artery angiogram showed a small defect in the proximal portion of the right CCA and a pseudoaneurysm, for which a stent graft was planned. A JOSTENT (length 28 mm, diameter 6 mm, JOMED, Rangendingen, Germany) was inserted and deployed over the CCA. After placement of the stent, a carotid artery angiogram showed no blood flow to the pseudoaneurysm (Figure 4). After three months, a neck ultrasound revealed no blood flow to the pseudoaneurysm and indicated that the blood flow of the CCA was intact.

## 4. Discussion

The causes of a pseudoaneurysm are varied but are most commonly associated with blunt or penetrating trauma. Other cause includes iatrogenic origin, inflammation, infection, vasculitis, tumor, and arteriosclerosis; however, there are also cases of unknown origin. The common causes of vasculitis resulting in a pseudoaneurysm include Takayusu's disease, polyarteritis nodosa, Kawasaki disease, and Behcet's disease [3].

In our cases, the first patient suffered from Behcet's disease, where his vasculitis appeared to be the cause of the CCA pseudoaneurysm. The patient in the second case had no history of trauma or any other specific disease, and thus the cause of the pseudoaneurysm was unknown.

The main symptoms of Behcet's disease include uveitis, oral ulcers, and genital ulcers. The rate of vascular involvement is between 23 and 62%, and approximately 95% of vascular involvement occurs in the venous system. Compared to the venous system, involvement of the arterial system is quite limited (around 2.2–7.7%), and while any artery has the potential to be affected, the most commonly affected arteries are the aorta, the pulmonary artery, and the femoral artery [4, 5].

There are two forms of pathogenesis that may result in a pseudoaneurysm. The first form is due to thinning of the tunica media, which can lead to rupture of the internal or external elastic lamina. The second form is vasculitis with lymphocytic infiltration in the area of the vaso vasorum [5].

The conventional treatment for pseudoaneurysm is resection and placement of a prosthetic or an autogenous vein graft. Although surgery is an effective treatment method, it is a technically complicated procedure that requires much experience, and complications such as cranial nerve palsy, stroke, rupture during surgery, or leakage into the surgical area can occur. The rate of stroke or death during surgery is reported to be between 9 and 15%, and the incidence of cranial nerve injuries is reported to be as high as 15%. Furthermore, in patients with Bechet's disease, surgical options tend to be limited due to the potential for skin and connective tissue inflammation [6, 7]. Recently, endovascular insertion of stent grafts has been developed. In contrast to surgery, general anesthesia is not necessary with a stent graft, thereby allowing for neurological monitoring during the procedure. In addition, since highly problematic areas are more easily treated, faster care is possible [8]. Park et al. reported that stent grafts are a safe and effective way to treat aortic and arterial aneurysms in Bechet's disease, where the patency of stent grafts were maintained in six of seven patients (86%) [9]. In addition, successful treatment of a CCA pseudoaneurysm with a stent graft has also been reported [10]. Stent grafts are also used to prevent carotid artery rupture due to cancer recurrence in the neck.

The JOSTENT graft is composed of a polytetrafluoroethylene graft material sandwiched between a stainless steel balloon-expandable stent. Polyfluoroethylene, a suitable material for placement into the body, has long been used in vascular prostheses [6]. Uncovered stents are also effective for treatment of a dissecting aneurysm with an intimal flap or small defects in the arterial wall. In cases of wide-necked aneurysms, flow into the aneurysm is difficult to exclude with an uncovered stent, and thus coil embolization of the aneurysm sac is often necessary. Today, wide-necked aneurysms or pseudoaneurysms can be more appropriately treated with a covered stent, leading to immediate and definitive reconstruction of the arterial wall [10, 11].

Possible complications of stent grafts include microembolism or vascular rupture. In our first case, an intracranial arterial microembolism occurred immediately after placement of the stent graft. According to the literature, the incidence of microembolism resulting from a coil embolization of an intracranial artery aneurysm is 8–28%, which can be treated by intra-arterial infusion of nonpeptide Gp llb/llla antagonist [12].

In conclusion, the possibility of a pseudoaneurysm must be considered when a patient complains of a pulsating neck mass, even if there is no history of trauma, as was the case for the two patients in this study. Based on our results, stent grafts can be an effective alternative to surgery for the treatment of CCA pseudoaneurysms.
